# The Administration of Anesthetics1Read at the meeting of the Obstetrical and Gynecological Section, Buffalo Academy of Medicine, September 26, 1899.

**Published:** 1899-12

**Authors:** Frank W. McGuire

**Affiliations:** Buffalo, N. Y., First assistant to City Hospital for Women


### THE ADMINISTRATION OF ANESTHETICS.[1]

By FRANK W. McGUIRE, M. D., Buffalo, N. Y.,

First assistant to City Hospital for Women.

I THINK all will agree that this important subject does not receive the attention it should from the medical profession, when we consider the number of anesthetics that are given and the limited few who can skilfully execute this task. Will anyone deny that he who undertakes such a task should be thoroughly informed on this subject? How often we see an anesthetic given where the patient at the beginning is struggling from the too rapid administration, or where it has taken an excessively long time to obtain complete anesthesia, and often, when it is reached, the patient is not kept there. Not infrequently, too, we find the anesthetiser much perplexed, not knowing whether to allow more air or more anesthetic, his rule being to give more anesthetic if the patient moves, if quiet to let them alone.

The administration of an anesthetic is a task that we all have to perform on occasions and it seems to me that we are playing with death at long odds, if we do not know whether a patient should receive more anesthetic or more air. We all know, as students, that every operable condition, many of which the physician is never called upon to perform, is used to the best advantage to impart knowledge, except in the case of the administration of an anesthetic, but we hope that the teachers in our medical colleges will some time in the near future devote more time to the practical teaching of this important subject, availing themselves of every opportunity to impart knowledge upon it. It appears to me that the hospital clinics are the proper places for acquiring knowledge on this subject and, if properly conducted, would turn out better anesthetisers. A student will learn more in administering one anesthetic under the direction of a competent instructor than by the administration of several, if left to his own resources. At present a student acquires his knowledge and skill by the hardest kind of experience and at an entirely unknown expense to his patient.

Some look upon anesthetising as a menial position, but a skilful administrator of anesthetics is a respected personage among his fellow practitioners, holding a responsible position, being second only to the operator. These conditions will justify a paper on this subject with special reference to the needs of the student, who wishes to

---

1. Read at the meeting of the Obstetrical and Gynecological Section, Buffalo Academy of Medicine, September 26, 1899.

be able to give an anesthetic in a skilful manner, with safety to his patients and satisfaction to the surgeon.

### PREPARATION OF PATIENT.

A few days' preparation is a great advantage. The heart and lungs should be examined for any pathological condition. The urine should be examined for evidence of nephritis. The amounts of solids and urea should be ascertained. The intestinal canal should be cleared out by a brisk cathartic. Two hours before the operation the lower bowel should receive a flushing. Some advise the retention of a pint of water in the rectum, in order to lessen the thirst that follows operation, while others advise the drinking of half a pint to a pint of water two hours previous to operation. Either of these seems to me to be of questionable utility, and should not be used in operations on uterus or bladder, as both predispose to a full bladder. The latter should not be used in any operation, as anything taken into the stomach so recently before anesthesia predisposes to vomiting. A much better means of accomplishing this end is to give an enema of four ounces of a saline solution every two hours as soon as consciousness is restored.

During this period the patient should receive a light nourishing diet. If the operation is performed during the forenoon, the patient should receive no breakfast, especially if the operation is in the peritoneal cavity; and if during the afternoon, no dinner should be eaten. At least five hours should elapse between an operation and the taking of any food into the stomach, especially by nervous patients. Strychnine, gr. 1-30, hypodermatically, every four hours is a good safeguard. It is important that the anesthetiser should obtain, if possible, the confidence of the patient. If he does not know the patient, he should be introduced by the family physician, who should assure the patient that he will be in skilful hands. The anesthetiser should then talk to the patient for a few moments, ascertaining that the mouth is free from foreign bodies. By this you impress the patient of your carefulness. Some vaseline should be rubbed on the lips, cheeks and nose, to prevent irritation from the anesthetic. The patient should be told to keep the eyes closed. Examine the arms for ankylosis, as an unnoticed fixed elbow may cause one to believe that the patient is not relaxed, and consequently the anesthetic may be pushed too far. See that the clothing is easily removable and no unnecessary clothing is worn. If tight about the chest, abdomen or throat, it will interfere with the respiration and circulation. The patient should lie in a horizontal position, the head only elevated to a comfortable breathing posture; arms should be folded, so that the end of the sleeve can be pinned to the shoulder, unless in the way of the surgeon. Hands above head, on account of raising the clavicles, predisposes to an excessively full right heart from the return circulation. An anesthetic should not be given on a lounge, as we are sometimes asked to do in private houses, as the head is elevated too much, or if for a dentist see that the chair is in a horizontal position and when the patient has sufficient anesthetic the head of the chair may be slightly raised.

### DROPPERS, INHALERS AND SO FORTH.

For chloroform I find that none compares with a two ounce bottle, with glass stopper, a notch being in the stopper and neck of bottle, so that, when placed opposite each other, a drop at a time will escape. Patent droppers are hard to regulate and often allow too much anesthetic to escape at one time. The inhaler should be a common wire mask, covered with one thickness of old linen, made from old tablecloths or napkins, or two thicknesses of cotton gauze. Linen being more absorbent than cotton allows the mask to become wet; on this account the cotton is to be preferred. A fresh one should be used each time, or the old one sterilised, as disease has been contracted from contaminated inhalers. For ether, a towel about a folded paper, with absorbent cotton in the cone, will answer as well as any of the elaborate patent inhalers. Its cleanliness and simplicity is much in its favor, but in the hands of a careless anesthetiser a patient may be readily asphyxiated, which detracts from its usefulness. Besides the apparatus already mentioned, there should be ready for use in every well-equipped operating-room the following—namely, a strong tenaculum, sponges upon holders, a mouth gag, a hypodermic syringe with strychnia, a cylinder of oxygen, with apparatus for its administration, an electric battery and a Fell's artificial respiration apparatus. Atropine should also be at hand to be used as a stimulant to the heart and respiration, to overcome the great fall of arterial pressure which occurs in serious collapse and heart syncope.

### QUALIFICATIONS OF ANESTHETISER AND PREPARATION.

I believe if one of us required an anesthetic we would use as much care in selecting the anesthetiser as we would the surgeon, unless an operation was to be performed that required except iona skill and experience. Then why should we not use the same care in selecting the anesthetiser for our patients, and not trust the task to a student, nurse or some physician who is unfamiliar with its administration? The anesthetiser should be cool, thoughtful and above all should have experience: one who appreciates the great responsibility that rests with him, giving his undivided attention to the anesthesia and not devoting, as we frequently see, almost his whole attention to the operation. He should be clean. Having cleansed his hands, he should don a sterilised suit and cap. After operation he should see that the patient is properly placed in bed and watch his condition until he regains consciousness. The patient should not be allowed a pillow for some hours after operation. If a laparatomy, not until the bowels have moved and all vomiting has ceased.

### CHOICE OF ANESTHETICS.

We should know that we have a pure drug, as many of the distressing symptoms are produced by impurities, especially the compounds of methyl and their decomposition products. (These are free chlorine compounds of hydrocarbons with chlorine, formic and acetic acid, and the like.) I believe the simplest and most practical is "Hepp's smelling test," which is made by dipping chemically pure filter paper into the chloroform and allowing it to evaporate. If the chloroform is pure, there will be no odor, but if it is impure, there will remain more or less of a sharp, irritating and unpleasant odor. Chloroform when pure is a clear, colorless, volatile liquid, having a pleasant, aromatic odor and a sweetish taste. It should be kept in well-stoppered, dark colored bottles and bought in quantities that will ensure its not becoming old. It has been recommended that the addition of half to 1 per cent. of absolute alcohol will prevent decomposition and will not enhance its value as an anesthetic. I do not intend to enter into the discussion of chloroform vs. ether, as I believe we all agree that chloroform is the best general anesthetic we have at the present time. There are undoubtedly more reported deaths from chloroform upon the table, but a number of deaths occur from pneumonia, bronchitis, suppression of urine and nephritis, as the result of the administration of ether, but are never charged against it. Chloroform is given in preference to ether in the following conditions—namely, in all pulmonary diseases, in renal disease, in disease of the bloodvessels, in abdominal distention, in cardiac lesions, unless compensation is disturbed, in cases of severe pain, in operations by gaslight, in obstetrical cases, in cranial operations, in laryngeal operations, in abdominal operations, in operations on the eye, face and oral cavity, in children and old people, in emergency cases where speed is required, in persons known to bear ether badly, and where the anesthetiser is skilled. Ether may be given in preference to chloroform in the following conditions—namely, in cases where the patient is known to bear chloroform badly, where the anesthetiser is unskilled, in extreme prostration, in collapse, following loss of blood, in chronic alcoholism, and when patient has taken chloroform for some time, face blanching frequently, pulse (130 to 150) showing that chloroform is affecting the heart and vessels. Often the substitution of ether will do away with these untoward symptoms from the stimulation it produces. Never give any anesthetic in operations for the repair of the soft parts following labor, as it is almost certain to produce alarming hemorrhage.

### THE ADMINISTRATION OF THE ANESTHETIC.

The anesthetic should be given slowly, but persistently, holding the cone from four to six inches from face, allowing the patient to breathe a few inhalations without any anesthetic on the cone. Then drop on one or two drops, requesting the patient to breathe naturally and regularly. Patient will frequently breathe regularly, if told to count. Gradually allow the cone to come nearer the face until it rests on it, slowly increasing the quantity on the cone according to the amount the patient requires. I do not believe that any set rule can be used for increasing the anesthetic, as some can take much larger quantities than others without experiencing any unpleasantness. Experience teaches the proper amount. If a patient should cough or struggle, remove the mask and allow a few respirations without any anesthetic. In this way patients are frequently anesthetised without any unpleasant sensations. To recapitulate, I wish to impress the fact that the process should commence at zero, and the strength of the vapor be gradually increased to the amount necessary to produce the required effect. This is the safest and most scientific mode. When the desired effect is produced, the patient should be kept there—if you will allow the term—"his head just above water." If you allow him to come from under the influence of the anesthetic and then push him under again, now up, now down, then he is in imminent danger. When once under, except in rare cases, if he recovers so that in your hurry to get him relaxed you pour on a larger quantity, or soak the mask, the fault is yours and the subsequent danger that may arise should be charged to your negligence and not to chloroform.

### STAGE OF ANESTHESIA.

(*a*) First stage of stimulation is of very brief duration and consists of a heightening of all the senses, a mild form of intoxication, which rapidly and unconsciously passes into the second stage. (*b*) Second stage—stage of excitement, stage of struggling. In this stage there is the conscious or perhaps unconscious effort of the individual in a stupefied state to fight against some of the symptoms produced by the anesthetic. We frequently have produced spasm of the glottis from the irritating effect of too highly concentrated vapor, but where the drug is given very cautiously in weak, delicate persons, or in those whose whole attention is directed to other interests, as during parturition, the second stage may pass into the third without any resistance. In strong, robust people and alcoholics the excitement is generally marked. During this excitement great care is necessary, especially with children. If the vapor is too concentrated, enough anesthetic may be inhaled with one inspiration to paralyse the heart. The third stage— (*c*) stage of surgical anesthesia. In this stage all the reflexes, except those of the involuntary muscles become abolished, the muscles are relaxed, the respiration slower and fuller and the pulse diminished in force and frequency. By watching the reflexes carefully and adding enough anesthetic to barely keep them abolished, a patient may be kept in this stage for hours. If the anesthesia is carried still further, we get the fourth stage—(*d*) the stage of paralysis. The face is suddenly blanched, or may be cyanotic, veins distended, respirations slow, shallow, sighing and, finally, ceasing. Pulse rapid, feeble, sometimes fluttering and may stop without warning. Pupils are widely dilated, eyes set and rigid, and death rapidly ensues unless prompt means of resuscitation are used.

### PHYSIOLOGY OF CHLOROFORM.

Its exact physiology is still a matter of dispute, which, to say the least, is remarkable after fifty years of the use of the drug, which has been the greatest boon to humanity since the introduction of opium. Most observers agree, however, that it always causes a marked fall in blood pressure, with slowing of the heart-beat and respiration, the latter finally ceasing after a sufficiently large dose has been administered. This fall in blood pressure, has been variously explained as being due either to vaso-motor depressions produced centrally, or by weakening the heart's action. Both factors probably play a part in producing it, but the latter is most to be feared, the drug acting as a direct poison to the heart muscle.

Experiments on lower animals show that when chloroform is pushed until it produces death the respiration ceases first, which is the cause of death. On the other hand, in the majority of reported fatal cases in the human being, the heart has stopped first and in some instances respiratory efforts have been noted some minutes after the heart ceased beating. Under such circumstances, I think the best view to take of the matter is that chloroform, when given in poisonous doses, usually kills by paralysing the respiratory center, but that it has a direct toxic effect on the heart muscle and under certain conditions, not at present understood, can cause death before large enough doses have been absorbed to destroy life in the usual way. Upon post mortem, in this latter condition, the heart is found dilated and its cavities filled with fluid blood. Failure of the heart, in this way, may be gradual, but often it stops suddenly without warning, and resists all attempts at resuscitation. Death has also been reported as occurring from fright when only a few minims of the drug were inhaled.

### REFLEXES.

Our object should be to give the least possible anesthetic, but sufficient to keep the patient in the third stage, and I believe this can be accomplished more skilfully by watching the ocular reflexes than by any other. The pupils at the beginning are usually dilated, unless morphine has been administered previously. They will usually contract under chloroform and should remain so while safe and efficient quantities are administered. Whereas, if patient is allowed to recover somewhat, they will dilate. The pupils will also widely dilate when chloroform is pushed to the stage of paralysis. It is just at this time that it is very important to know whether you should give your patient more chloroform, or allow him more air. I am always directed in this by feeling the rigidity or tension of the eye—either external to eyelids, or on cornea if necessary. If the pupils are widely dilated and the eye rigid, it is time to give your patient a liberal allowance of air. If the pupils are contracted and the eyes soft and flabby, give more chloroform. Another important reflex is the oscillating edge of the iris. While this remains the anesthetic can be safely pushed, but when it disappears the point of danger is usually approaching.

There is a certain number of patients whose pupils remain dilated during the entire anesthesia, especially if it is pushed too rapidly at the beginning. Another class embraces those in which the ocular reflexes do not respond. In these you have to be guided by the remaining reflexes, the condition of the heart and the condition of the respiration. If any do not bear the chloroform well, ether should always be substituted. In using the ocular reflex always examine for a glass eye, as serious mistakes have already been made by not noticing this condition. There are objections to using the ocular reflexes, as conjunctivitis and ulcerations of the cornea have been caused by either an unclean finger, with a long nail, or by a drop of chloroform. The former can be prevented by cutting the finger nails short and having hands clean; the latter by using a towel over the eyes, and only removing it when you wish to examine the eye. I believe these objections count for *nil*, as compared with the safety of our patients.

Muscular relaxation may be tested from time to time by the raising of the forearm. Snoring and stertorous breathing indicate pharyngeal and faucial paralysis and with this the state of surgical anesthesia. The masseter muscles are generally the last to relax. If, during anesthesia, the patient gains control of the jaw, he is coming out of the influence of the anesthetic. In operations for suppurating appendicitis, pyosalpinx, intestinal obstruction, where more or less peritonitis is present, also in separating old adhesions, the patient may be profoundly anesthetised and the abdominal muscles will be still unrelaxed. This condition can generally be obviated by giving a hypodermic of morphine before the anesthetic is commenced. It must not be forgotten that reflexes are produced in operations on the prepuce, rectum and vulva when patient is under complete anesthesia. Also in hysterical women, the fingers will sometimes move and, if you depend entirely on this reflex and push the anesthetic, you will soon have your patient in a condition in which the respiration is embarrassed or has ceased and the pulse becoming imperceptible. In other words, you will have the stage of paralysis.

### THE CIRCULATION.

The color of face, nails and lips should be watched carefully, as blanching of these parts may occur before any trouble is indicated in the blood current. The pulse at the beginning is generally accelerated from fear, but when the third stage is reached it generally falls to normal and remains there. The anesthetiser should know for himself the condition of the pulse and should never trust it to some one else. The pulse may sometimes be intermittent at the beginning, which may be caused by fear or a tobacco heart. A pulse of this kind should be watched carefully and, if any untoward symptoms arise, ether should be used. If it is caused by fear, the heart will become regular when surgical anesthesia is reached. Many variations of the pulse may exist without serious consequences arising, but the most assuring one to the anesthetiser is the regular and steady one. In the latter stages when paralysis is impending from an excess of chloroform, you may find the pulse weak, rapid and irregular. It may also be very soft with no force, or may become suddenly imperceptible.

### RESPIRATION.

The character of patient's breathing is probably the most important sign of his condition. The ear rather than the eye should be depended upon for the assurance that he is breathing properly, as the chest may rise and fall regularly and no air enter the lungs. The sound of the air entering the trachea and bronchial tubes is unmistakable and the ear readily detects it. I do not wish to infer that the anesthetiser should concentrate his attention to the breathing or to any one point, but his ear should be so well trained as to detect the slightest change. Snoring and stertorous breathing is produced by paralysis of the pharyngeal and faucial muscles, and is indicative of profound narcosis. With ether this is not necessarily a dangerous condition, but when it occurs during chloroform narcosis it should always receive attention, ascertaining the profoundness of the anesthesia. Turning the head a little to one side, or from one side to the other, will frequently stop it. If not, the lower jaw should be brought forward.

Patients with adenoids and nasal obstructions, or those who are fleshy, may have disagreeable respiration during the entire anesthesia. The respirations at the beginning of the anesthesia are slow, but the patient should be directed to breathe naturally. During the stage of excitement patients will frequently hold their breath for some moments and then take a full deep one. At this period the choroform should not be pushed, as sufficient of the vapor may be inhaled with one deep breath to paralyse the heart muscle. If it is necessary to add more anesthetic, it should be done slowly and very carefully. When full anesthesia is reached the respirations are about normal, but if the stage of paralysis is reached, it becomes slower, more shallow and finally ceases. During anesthesia, short, jerky and rapidly increasing respirations, repeated efforts at deglutition, with pulse increasing rapidly, dilated pupils responding to light, are all symptoms which indicate that vomiting is approaching. If this condition is not noticed at once, and more anesthetic not given, you will have to undergo the annoyance of having your patient vomit.

The management of the tongue is very important. The more skilfully this is managed, the more skilful will be the anesthetiser. If the epiglottis closes, it should be brought forward by closing the mouth and bringing the inferior maxilla forward and, if necessary, slightly extending the head. Sometimes merely closing the mouth will remedy this condition. When the mouth is closed it should be known that there is no nasal obstruction. If necessary a catheter may be passed through nares. If this does not remedy the condition, use a strong tenaculum to bring tongue forward, or pass a needle with strong silk from side to side through the tongue and make traction. A tongue forceps, such as we usually see in the clinic should not be used, as it is unnecessary and mutilates the tongue, so as to be a great source of annoyance to the patient while convalescing. I believe this instrument should be relegated from every operating room. The tongue seldom requires bringing forward if the anesthetic is skilfully given. If a gas jet is burning in a room where chloroform is used, proper ventilation should be provided, as the fumes break up into acetylene and chlorine gases which are irritating to the throat and lungs and very depressing to the patient. The respiration should not be embarrassed by leg supports which pass around neck and shoulder. The best supports are those which are attached to the table, or another kind may be used in which a belt passes around body at umbilical region and legs attached to band by strap.

### STIMULANTS.

The indiscriminate using of stimulants is a harmful practice and one that should be discouraged. Frequently patients are stimulated when it is entirely unnecessary. If the pulse in an adult should reach 130 to 150, the patient should be permitted to come from under the anesthetic a little or, if you please, "kept a little closer to shore," and allowed more air. In doing this, oxygen is introduced into the system, one of our best stimulants. This will frequently bring the pulse down to 90 or 100. If the pulse still keeps up under anesthesia, chloroform being used, resort to ether. This change will sometimes work wonderfully well. If it is still necessary to use a stimulant, strychnine, 1-30 to 1-20, repeating if necessary, may be administered. If the circulation is much depressed, use atropine.

This contracts the arterioles and veins. Digitalin is also a good stimulant. Hot injection of normal saline solution into the peritoneal cavity or by bowel or transfusion may be employed. Heat over heart area is a convenient and very useful remedy. Nitrites or alcohol should not be used in deep narcosis, as they will increase the difficulty.

### RELATION OF ANESTHETISER TO OPERATOR.

If he is inexperienced, he will need instruction and be grateful for it. Occasionally he will need reprimanding and possibly censure. If so, he should take them silently; but, let it be remembered, he does not need abuse. If the surgeon so far forgets himself as to resort to it, the anesthetiser had better resign his position to some one who, possibly, will not object to abuse. A man in any position can command respect, and if he does not, the fault is his own. Some operators delight in using caustic remarks—often from habit—and frequently without cause. This should not confuse one, as neither you nor your patient can afford it. The anesthetiser should have the entire confidence of the surgeon, and, in order to possess it, he must be skilled. No person appreciates a competent anesthetiser more than the surgeon who wishes to concentrate his whole thought and attention on his work. It is almost impossible for him to do good work, if he has to be continually on the lookout for his patient's condition. The anesthetiser should know more, or at least as much about anesthesia as the surgeon. He should always know the condition of his patient and when questioned regarding him, should answer in no uncertain tone. Often the tone of his voice will indicate whether he can be depended upon or not.

### CONCLUDING REMARKS.

I have purposely left out the accidents that occur during anesthesia and their treatment as it would make my paper too long and besides they can be read in almost any text-book. Dr. Parmenter of this city has written an article, published in April, 1896, in the BUFFALO MEDICAL JOURNAL, in which he gives a prominent part to the accidents and their treatment. A good anesthetiser will give his whole attention to the patient, not following the steps of operation, or allowing anyone to converse with him. In trivial operations, we sometimes see a desire on the part of the anesthetiser to treat the anesthesia as trivial, but every anesthesia, no matter how trifling the operation, should receive undivided attention. All the indicators of the patient's condition should be carefully watched. Observe the color of the face, lips and nails, watch pulse, character of respiration, reflexes, oscillation of the pupils and rigidity of the eye. He should know his subject thoroughly and have his knowledge always available. He should keep cool, think carefully, decide wisely and act promptly.

An anesthetiser should be compensated for his work. I have known of cases where the operator received for his operation from $50 to $200 and the anesthetiser from $3 to $5. In fact, he is lucky if he receives anything at all. This is a fair example of how things exist today. It seems to me that when people are able to pay such prices for operations, they should also give the anesthetiser a reasonable compensation.

Finally, I have not attempted to present an exhaustive bibliographical article, but one which is purely clinical, and in closing cannot do better than reassert that upon no condition does the welfare of the patient depend more than upon the skill, ability and discretion of the anesthetiser, and that nothing is more fallacious than that an anesthetic can be given in the haphazard way we often see it done.

158 NORTHLAND AVENUE.

